# Vector Appropriate Distraction and Reduction of a Neglected Trimalleolar Fracture-Dislocation Performed with a Temporary Intraoperative External Fixator

**DOI:** 10.1155/2021/5577626

**Published:** 2021-07-10

**Authors:** Jordan Brand, Jay Moran, Brad Yoo

**Affiliations:** ^1^Yale School of Medicine, Department of Orthopaedics and Rehabilitation, New Haven, CT, USA; ^2^Yale School of Medicine, New Haven, CT, USA

## Abstract

Traumatic fractures of the ankle can occur with concomitant tibiotalar dislocations, necessitating complex treatment. These injuries have higher rates of loose bodies, open injuries, postoperative complications, and worse patient reported outcomes compared to ankle fractures without dislocation. Patients with neglected or delayed presentations are associated with even higher rates of postoperative complications and worse outcomes compared to acute injuries. The chronicity of the injury leads to soft tissue contractures and malunited fractures, obligating a care plan which involves gradual reduction with a multiplanar external fixator with or without internal fixation at a later date. We discuss a 60-year-old homeless man who presented four weeks after an open trimalleolar fracture-dislocation and was definitely treated with an acute one-stage procedure. Anatomic reduction and stable fixation was achieved through a lateral malleolus osteotomy, soft tissue releases, TAL, and a temporary intraoperative external fixator. This technique was advantageous in this instance of anticipated patient noncompliance. We advocate for the judicious use of the described technique in similar challenging situations.

## 1. Introduction

Ankle fractures are the most common fracture of the lower extremity, accounting for approximately 9% of adult fractures [1]. In these fractures, concomitant tibiotalar dislocations have been reported in 21% to 36% of cases [2]. Such fracture-dislocation injuries are associated with higher rates of open injury, chronic pain, intra-articular loose bodies, posttraumatic osteoarthritis, and worse patient-reported outcomes when compared to isolated ankle fractures [2].

Neglected, chronic fracture-dislocations are associated with increased risks of infection, amputation, and impaired functional outcomes, requiring more specialized treatment plans than acute fracture-dislocations of the ankle [3, 4]. Few reports exist in the literature describing the treatment and outcomes of these chronic injuries, and when encountered, it can be technically challenging to spare the ankle joint due to soft tissue contractures and malunited fractures [4–7].

This report presents a 60-year-old homeless man who sustained an open fracture-dislocation of the left ankle and presented four weeks later. The patient was treated with a single-stage procedure using a vector appropriate distraction and reduction to address this complex and underreported presentation.

## 2. Case Report

A 60-year-old homeless male presented four weeks after his left ankle was twisted in an altercation. At that time, he experienced immediate deformity and the inability to bear weight. The patient decided to delay treatment until he was able to place weight on his leg and seek medical attention.

**Figure 1:**
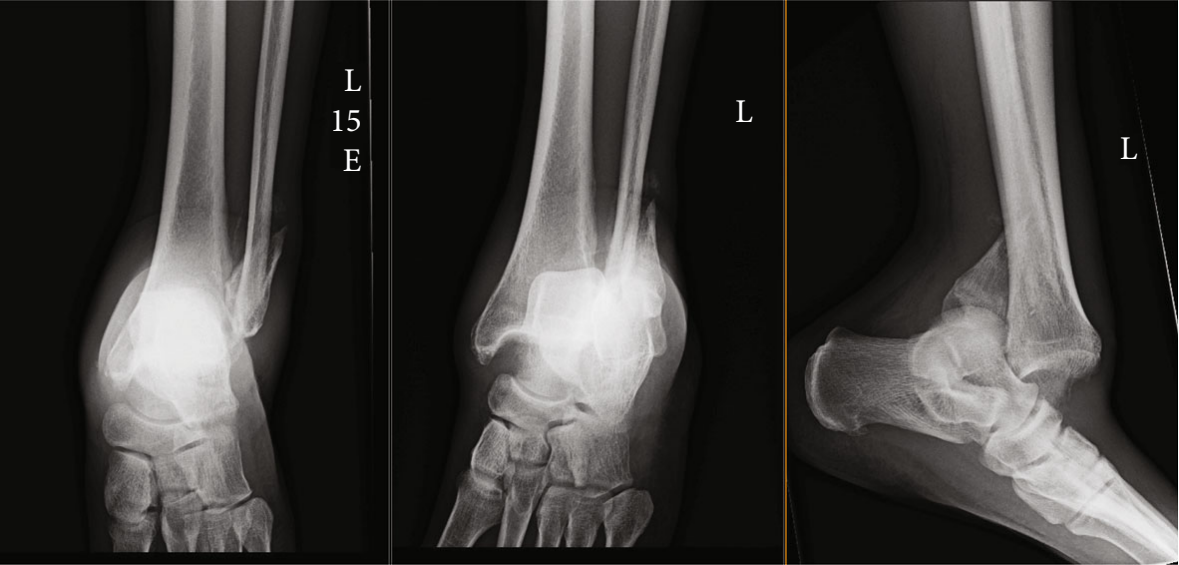
AP, mortise, and lateral radiographs at the time of initial presentation showing the neglected fracture-dislocation of the ankle.

**Figure 2:**
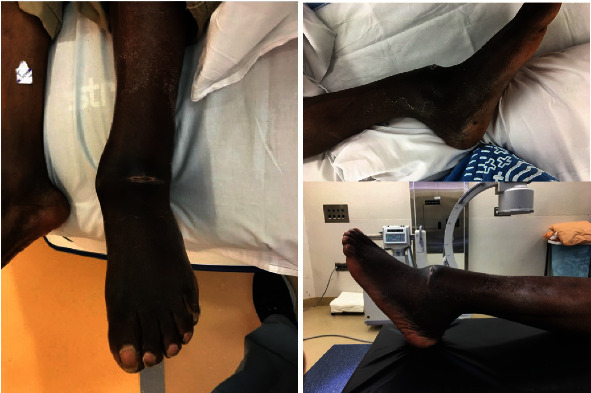
Clinical photos of the patient's ankle prior to surgery showing open ankle dislocation.

Upon presentation to the emergency room four weeks later, the following clinical and radiographic appearance was documented (Figures 1 and 2). Ankle imaging demonstrated a chronic dislocation, with typical posterior translation of the talus with respect to the tibia, along with concomitant posterior translation of the distal fibular segment (Figure 2). He maintained approximately 10 degrees of motion through his tibiotalar joint. Due to the deformity, the patient stated that his lower extremity was not functional for simple activities of daily living, such as prolonged standing or walking any distance less than a block. Although he would comply with postoperative nonweight-bearing restrictions, the patient stated he would not participate in any care plan which required more than one surgery. Based upon prior behavior, the care team judged the patient would be unable to maintain or adjust a multiplanar corrective frame, nor return for periodic office visits and radiographs. As such, a single-stage procedure was planned.

The patient was brought to the operating theater with general anesthesia, pharmacologic paralysis, and supine positioning. Two approaches were used. The first was a lateral longitudinal incision along the anticipated position of the posterolateral fibular border once reduced. Following fibular exposure, osteoclasis of the developing fracture callus was performed. As the distal segment was mobilized, it became apparent that the syndesmosis was grossly incompetent. The distal fibular segment was externally rotated, and the syndesmotic incisura was debrided of reactive tissue. Through this interval, the posterior capsule was released off its pathologic attachments to the posterior tibia.

An anteromedial approach was performed to access and debride the medial joint. The anterior and medial capsule were released, yet the talus remained incarcerated within the posterior tibia. Manual manipulation failed to mobilize the talus. A fibular reduction, which typically helps the talar translation, was not feasible as the dislocation was too rigid and displaced. A tendo-Achilles lengthening (TAL) was performed in attempt to translate the talus more readily, but this did not permit complete relocation.

A temporary intraoperative external fixator was applied to effect the relocation. Two 5.0 mm Schanz pins were inserted medial to lateral in the tibial diaphysis and connected with a carbon fiber rod. With a rod-to-rod connector, another carbon fiber rod was secured perpendicular to the first rod. A 5.0 mm Schanz pin was inserted in the medial talar body. A long carbon fiber rod was now positioned with a proximal posterior to distal anterior orientation, being secured proximally to the drop-down tibial pin cluster and secured distally to the medial talar pin. This would create a point of origin for distraction posterior to the tibia. To reinforce the tibial distraction construct, a triangular frame was constructed between the tibial pin cluster and the long distraction rod.

**Figure 3:**
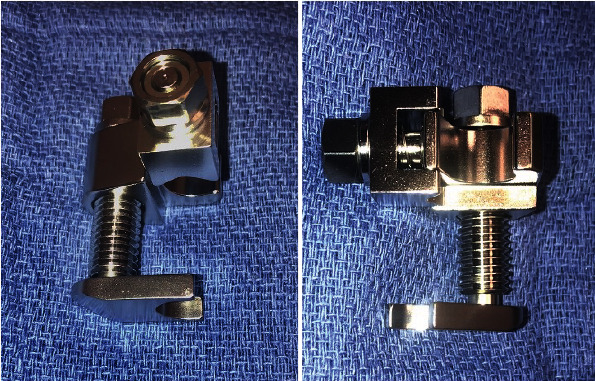
Photographs of the Open Compressor device (Synthes, Paoli, PA). The device's foot plate is at the bottom of each figure.

**Figure 4:**
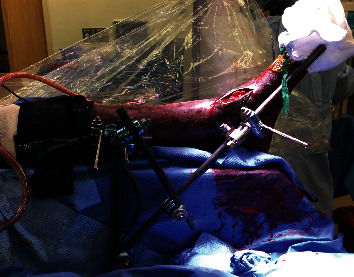
External-fixator construct used to facilitate reduction of chronic ankle dislocation.

An Open Compressor device (Synthes, Paoli, PA) was placed abutting the rod-to-pin connector nearest the talus (Figure 3). The clamp affixing the long rod to the medial talus was tightly secured to the medial talar Schantz pin, but not the carbon fiber rod. This would permit translation of the rod with respect to the medial talar pin bar clamp. The final construct is depicted in Figure 4.

**Figure 5:**
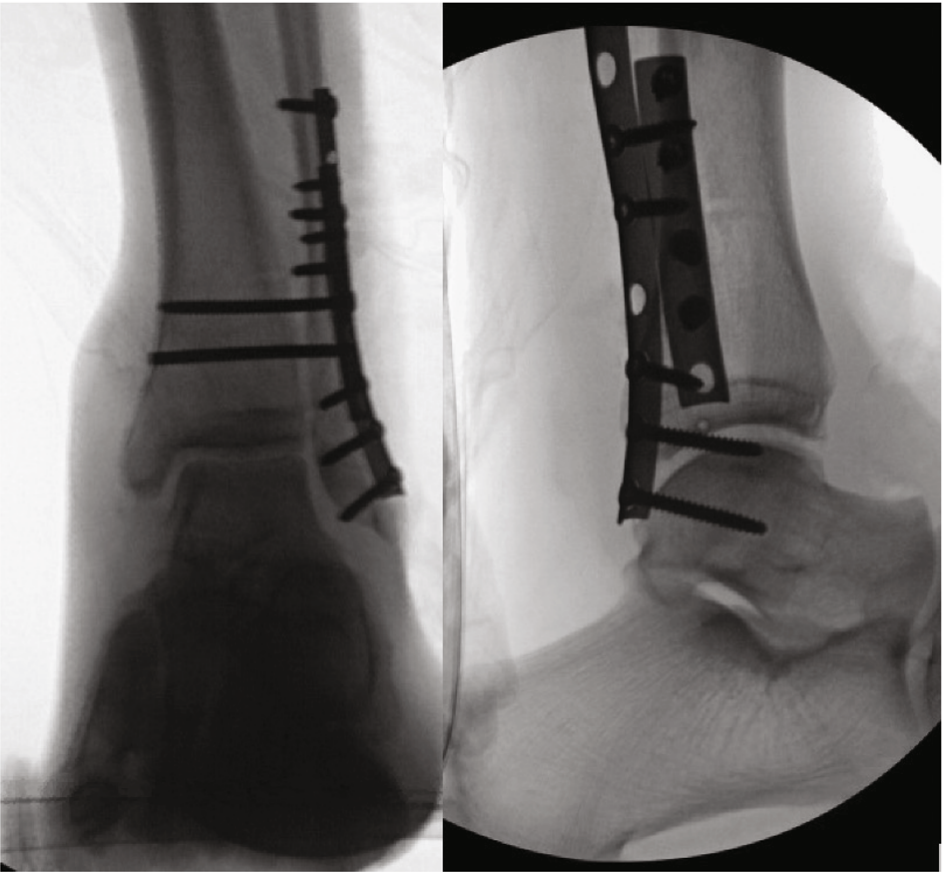
Final intraoperative imaging.

Linear translation of the talar pin was accomplished with the Open Compressor device. As the carbon fiber rod was oriented proximal posterior to distal anterior, so too was the direction of distraction and hence the translation of the talar body. Following anatomic reduction of the talar body convexity under the tibial plafond concavity, the fibula reduction was markedly improved. Fibular osteosynthesis was performed following fibular clamp reduction by two 1/3 tubular plates placed posterolaterally and laterally on the fibula. Syndesmotic reduction was performed through an open visualization of the fibular reduction within the incisura and placement of quadricortical syndesmotic screws [8, 9]. The external fixator was then removed, and the reduction was confirmed as stable. Final intraoperative radiographs are depicted in Figure 5. After wound closure, the patient was placed in a short-leg splint and instructed to remain nonweight-bearing to this extremity.

**Figure 6:**
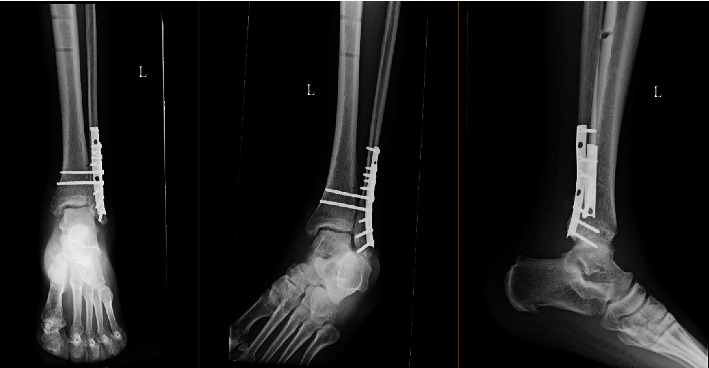
Radiographs approximately 3.5 weeks postoperatively.

The patient returned to clinic two months after surgery, at which point weight-bearing was initiated. Radiographs confirmed an appropriate reduction (Figure 6). He returned again 3 months after surgery at which time he had a 30° arc of motion at the ankle. He reported resolving pain and did not require assistive devices for ambulation. A telephone appointment was conducted one year after surgery. At this point, the patient was able to perform daily activities and reported not requiring assistive devices or analgesics.

## 3. Discussion

In North America, neglected fracture-dislocations of the ankle are uncommon [4–7]. In these cases, ORIF is difficult, as patients have developed soft tissue contractures, pathologic scarring, and malunion [4, 7]. This latter point is critical, as the talar position is inextricably linked to the position of the lateral malleolus. Fibular malunion is a common cause of mortise incongruity and talar instability and is the focal point of attention during reduction of a chronic dislocation [10–13]. Here, we show the reciprocal of this relationship to also be true. Even after lateral malleolus osteoclasis, the fibula could not be appropriately reduced given the mechanical block of the talus on the posterior tibia. Tibiotalar reduction was achieved and necessary to enable anatomic alignment of the lateral malleolus fracture.

Several surgical treatment plans have been reported in the literature for neglected fracture-dislocations of the ankle [4, 8, 14]. While the majority of acute ankle fracture-dislocations can be treated with single-stage ORIF, the soft tissue contractures and malunited fractures associated with neglected cases push surgeons to consider different surgical treatments [3, 7, 14]. Thomason et al. reported a neglected open ankle fracture-dislocation treated with a primary ankle arthrodesis due to the severity of the injury [3]. In that case, the patient presented five days after injury with an extensive soft tissue defect. The entire tibial plafond was grossly contaminated and had exited the skin medially. The tibialis anterior and posterior tendons as well as much of the articular cartilage were devitalized and thus removed at the time of the first surgery [3]. In contrast, our patient presented with a healed anterior wound, no evidence of an ongoing infection, and with significantly less cartilage damage.This prompted an attempt to spare the tibiotalar joint to preserve as much ankle mobility as possible.

For less severe cases than the one described by Thomason et al. [3], staged procedures are commonly utilized. Tellisi et al. used a minimally invasive two-stage procedure with an Ilizarov/Taylor Spatial Frame (TSF) to achieve gradual reduction and then ORIF [7]. Similarly, Shenoy et al. used a staged procedure in a case that required over a month of gradual reduction with a TSF [14]. These multistage procedures are the preferred treatment for neglected fracture-dislocations of the ankle, with a goal to gradually reduce the fracture with a TSF, minimizing iatrogenic soft tissue injury [7]. After gradual reduction is achieved, internal fixation is carried out on another date. A staged treatment option avoids aggressive soft tissue release and is more reliable than acute correction, as it allows for better reduction, minimizes damage to the soft tissue, and reduces postoperative complications involving infections or wound closure [4, 7]. These factors make the decision to choose staged procedures an attractive option over an acute correction for the majority of patients that present with neglected fracture-dislocations of the ankle. The use of staged procedures requires extensive patient follow-up, daily patient pin site care, and further surgeries to fully correct the pathology [7, 14, 15]. Additionally, the continued care associated with these procedures may incur higher healthcare costs, multiple implants, and prolonged treatment [15]. If continued care is neglected in-between procedures, the patient can have devastating long-term consequences and complications. Previous authors have suggested that staged procedures are contraindicated in noncompliant patients [15].

In acute correction of chronic fracture-dislocations of the ankle, the soft tissue contractures and malunited fractures that prevent traditional ORIF require extensive debridement [4]. Such techniques to treat fibular malunions are well reported and include osteotomy of the lateral malleolus and correction of the overall ankle and limb alignment [10–13]. Fibular osteotomy alone may not be enough to reduce the talus due to extensive soft tissue scarring, as was noted in our case [4]. Khan et al. could not achieve reduction with fibular osteotomy alone, requiring TAL with medial and posterior soft tissue releases [4]. In their case, the fibula displaced posterior to the syndesmotic incisura and became occupied by reactive scar tissue, requiring debridement and posterior capsule scarring in a shortened position to the posterior tibia [4]. Similarly, we performed a TAL, but the dislocation remained too rigid for reduction, necessitating the implementation of a vector-specific reduction device. This device allowed us to preserve as much soft tissue as possible, while simultaneously enabling us to achieve reduction and fixation acutely [4, 7]. As opposed to staged procedures, the extensive debridement required when acutely correcting this fracture-dislocation may increase vascular injury risk [4, 7]. In return, this may compromise the blood supply to the fracture site and increase the chances of AVN and future malunion [4, 7]. While the complications associated with acute correction can be more serious than those associated with staged procedures, it is important to consider patient demographics when choosing which surgical treatment option to use [7]. Treatment decisions must be made on an individual patient basis and can be the deciding factor for long-term success, especially in neglected fracture-dislocations of the ankle [4, 7].

In our case, the patient expressed inability to adhere with medical follow-up, anticipation of noncompliance, and poor access to medical care. The healthcare team and patient decided that an acute correction procedure to achieve both reduction and internal fixation was better indicated over staged procedures. As others have stressed, patient reliability and compliance must be considered when surgically treating this severe injury [3]. In patients that have trouble with compliance, the benefits of choosing an acute correction procedure may outweigh the potential risks associated with noncompliance in staged procedures. Arguably, staged procedures could have been used to gradually reduce the fracture and then internally fixate at a later date [4–7]. However, the acute reduction and internal fixation in the same setting enabled an earlier range-of-motion while simultaneously avoiding postoperative complications associated with compliance concerns. Additionally, our stepwise implementation allowed for anatomical reduction when ORIF could not be achieved from the start. Overall, this technique led to a successful outcome of a neglected fracture-dislocation of the ankle, and for specific patients, we advocate for its use in similar clinical situations.

## 4. Conclusion

Neglected ankle fracture-dislocations are uncommon injuries that require special surgical treatment. In our case, anatomic reduction and stable fixation was achievable via lateral malleolus osteotomy, soft tissue releases, TAL, and an intraoperative external fixator. While staged procedures are generally considered the most optimal treatment option for neglected ankle fractured-dislocations, acute correction may be indicated for a certain patient subset. In our case, a single-stage procedure led to a successful outcome of a neglected fracture-dislocation of the ankle and we advocate for its use in similar clinical situations.
